# Prediction Errors in Learning Drug Response from Gene Expression Data – Influence of Labeling, Sample Size, and Machine Learning Algorithm

**DOI:** 10.1371/journal.pone.0070294

**Published:** 2013-07-23

**Authors:** Immanuel Bayer, Philip Groth, Sebastian Schneckener

**Affiliations:** 1 Aachen Institute for Advanced Study in Computational Engineering Science (AICES), RWTH Aachen University; Aachen, Germany; 2 Therapeutic Research Group, Bayer Pharma AG, Berlin, Germany; 3 Systems Biology, Bayer Technology Services GmbH, Leverkusen, Germany; Dana-Farber Cancer Institute, United States of America

## Abstract

Model-based prediction is dependent on many choices ranging from the sample collection and prediction endpoint to the choice of algorithm and its parameters. Here we studied the effects of such choices, exemplified by predicting sensitivity (as IC50) of cancer cell lines towards a variety of compounds. For this, we used three independent sample collections and applied several machine learning algorithms for predicting a variety of endpoints for drug response. We compared all possible models for combinations of sample collections, algorithm, drug, and labeling to an identically generated null model. The predictability of treatment effects varies among compounds, i.e. response could be predicted for some but not for all. The choice of sample collection plays a major role towards lowering the prediction error, as does sample size. However, we found that no algorithm was able to consistently outperform the other and there was no significant difference between regression and two- or three class predictors in this experimental setting. These results indicate that response-modeling projects should direct efforts mainly towards sample collection and data quality, rather than method adjustment.

## Introduction

### Background

It is common clinical practice to treat cancer patients with a variety of therapeutic agents, due to the simple fact that drug efficacy varies among patients. Thus, general recommendations for drug selection and finding the optimal dosage for a certain drug are challenging tasks and often subject to change based on new standards of care. This is one of the main reasons why drug response to cancer therapeutics is being tested in *in vitro* studies, where cancer samples from different cell cultures or patients are cultivated under standardized laboratory conditions and tested for their reactions to a variety of compounds. In such laboratory environments, similar variance of response can be observed when cancer cells from different patients are treated with the same drug. Of course, it is expected that differences in drug response are closely related to the type of cancer under treatment. Other factors like ethnic, genetic and histological background of patients even with the same type of cancer play an important role in drug response, making cancer a very individual disease requiring a personalized treatment.

The toxicity of the medication as well as the severity of the disease itself requires a high degree of confidence in the effect of a drug for the specific patient. The field of personalized medicine is dealing with this type of challenge. One research focus is the identification of markers that can be used to individualize treatment recommendations, i.e. to reliably predict the response of cancer to drugs. Markers are selected among high-dimensional genetic data, such as gene expression data from microarrays and machine learning methods are used for the prediction.

However, there is a multitude of challenges given a fairly “simple” question (i.e. the prediction of drug response to cancer cell lines). The “no free lunch theorem” [1], for example, says that no predictive model is superior to another if the performance is averaged over all possible problems. This indicates that the search for a particularly good predictive model should be driven by the structure of the problem at hand. The data used in this study is high-dimensional genetic data from gene expression microarrays and has the special property that the number of variables p far exceeds the number of samples n. The major problems in dealing with such p>> n prediction tasks are extreme overfitting and high variation that can be observed in the fitted models. As a general guideline, the use of simple models or highly regularized approaches has been recommended [2].

### Study Aim

In this study, we have examined the factors influencing the feasibility of predicting the outcome of drug response experiments. An accurate prediction of drug response could help economize on the vast resources necessary for laboratory experiments. To this end, we have designed a study using well-known machine learning algorithms, response data of drugs inhibiting cell growth by targeting different mechanisms of the cell, gene expression data from in-house and public efforts. It is expected that the underlying biochemical processes of these cellular mechanisms can be differently well observed through gene expression analysis.

Therefore, we have formulated five hypotheses to determine important factors contributing to the above question.

Hypothesis relating to the choice of drug:

- H1: The successful prediction of efficacy depends on the particular drug.
  Hypotheses relating to the machine learning methods:
- H2.1: The machine learning algorithms used for the prediction task at hand have a strong influence on the predictability.
- H2.2: One labeling is superior in predictiveness than the others.
  Hypotheses relating to the choice of cell line panel:
- H3.1: There is a connection between the choice of panel and drug predictability.
- H3.2: The number of samples influences the predictiveness.

In this study, we have evaluated drug response predictions for 23 drugs. This prediction task has been solved by seven different machine learning approaches and up to three labeling variants (see Table 1). Thus, many different machine learning algorithms and gene expression drug response combinations have been evaluated in this study in order to generate data to determine the factors influencing the predictability of drug response in cell lines. Each combination of variables is fully defined by the notation:

**Table 1:** Overview of machine learning algorithms used in this study.

Algorithm	Regression	2-class	3-class
elastic net regression	RMSE	–	–
L1 PLR	–	AUC	–
PCA & random forest	RMSE	AUC	mAUC
PCA & SVM	RMSE	Accuracy	F1-Score
SVM	RMSE	Accuracy	F1-Score
random forest	RMSE	AUC	mAUC
two stage randomforest	RMSE	AUC	mAUC

The first column indicates the algorithm, the second to forth if this algorithm was applied to regression, binary or three class classification. A dash indicates that the algorithm was not used for that problem. Otherwise, the metric reported in this article is indicated. RMSE: Root mean square error. AUC: Area under the ROC curve, mAUC: multiclass AUC, F1-Score: this.



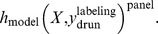



**Figure 1:**
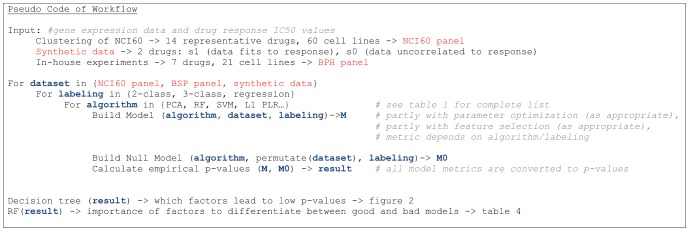
Illustration of main steps to calculate the p-value for model predictability. A model depending on algorithm, drug and panel is trained, used for prediction on a test set and the prediction results are evaluated by a metric. The same is done with a null model. Comparing the two evaluations results in an empirical p-value.

In the above notation, h_model_ can be any model according to Table 1 and *panel* can be any of the utilized cell line panels (see Materials & Methods). *Drug* denotes any of the 23 drugs considered, to which response was predicted using one of the three *labelings* (binary, i.e. responder vs. non-responder; ternary, i.e. responder, intermediate, non-responder, or continuous, i.e. the IC50 values of the drug). We have evaluated every possible combination of the variables for the original data and for null model data with randomly permutated class labels (illustrated in Figure 1). In total, 510 combinations have been evaluated and their empirical p-values have been recorded.

We utilized random forests (RF) and a decision tree for evaluating the prediction results and to identify factors influencing the prediction quality. We could show that the choice of the cell panel and the drug has a greater influence on the prediction quality than expected by chance. A correlation between small sample size and low predictive accurateness and high variation has been found. Thus, we could show that the efficacy of different drugs is not equally good to predict. The machine learning algorithm used for prediction has little to no influence on accuracy.

### Related Works

Brown et al. [3] compare five machine learning techniques to predict functional distinct gene classes by using gene expression data. They find that SVM outperform other techniques (i.e. Fisher’s linear discriminant, Parzen window, and tow decision tree learners).

Zhu and Hastie [4] compare support vector machine (SVM) and penalized logistic regression (PLR) for microarray cancer diagnosis problems. The techniques have been tested on three data sets. He finds that PLR and SVM perform similarly in cancer classification, though PLR with recursive feature elimination (RFE) often selects fewer genes. They stress PLR’s advantage to give class probabilities.

Diaz-Uriarte and Alvarez de Andres [5] compare machine learning algorithms by their ability to select relevant genes for sample classification. They use random forest to select features by iteratively fitting random forest and discarding features with smallest variable importance. They find that random forest have a comparable performance to other classification methods (diagonal linear discriminant analysis (DLDA), k nearest neighbors (KNN), and SVM) but achieve that with an often smaller set of genes.

Shi et al. [6] evaluate a large number of different models for predicting clinical endpoints in humans and toxicity endpoints in rodents. They report that predictivity depends mainly on the endpoint and on the experience of the modeling group.

Statnikov et al. [7] compare SVM and random forest for cancer diagnosis and clinical outcome prediction. They evaluate both methods with over 22 data sets. They report that random forests are outperformed by SVM with or without feature selection methods.

Riddick et al. [8] propose a multistep algorithm to predict in vitro drug sensitivity using gene expression data from the NCI60 panel. They build a predictive model for two drugs and test them on 19 breast cancer cell lines, which are not in the NCI60 panel. The three main steps in their research are: Selection of features based on random forest variable importance, removal of outlying cell lines based on the random forest case proximity, and training of random forest regression models on selected samples and features. They conclude that their algorithm is superior to existing techniques.

## Materials and Methods

### Working Hypotheses

Generally, two categories of machine learning algorithms exist: Regression models to predict continuous variables and classification models to predict categorical variables. The choice of the algorithm is then usually based on expert knowledge from the problem domain. A typical classification problem features two classes (binary classification) but some models can also be used for multiclass classification. The process of separating the cell lines according to the IC50 values into two or three classes is referred to as labeling here (e.g. responder vs. non-responder or responder vs. intermediate vs. non-responder). The factor labeling consists of three levels: regression, binary, and class3 (multi classification with 3 classes). All three levels have been applied, since there is no clear direction for a certain classification level suggested by prior research for the problem considered here. Different machine learning algorithms have been proposed [3], [4], [5], [6], [7], [8] as being superior to others to process microarray data. Considering all the aspects described above, different models for regression and classification have been selected for this study.

A panel of cell lines has different properties that can be expected to influence the prediction task. The cell line panels used in this study have differing numbers of samples (either 22 or 59) from a diverse range of tissues and cancer types. Therefore, it was tested whether the average predictiveness over all drugs is significantly different between the cell line panels. In such a case, it can be further tested whether the number of samples can explain this. Therefore, a model performing well on the larger panel and worse on the smaller panel is reevaluated with only a subset of the samples.

### Cell Line Panels

The most comprehensive study of compounds in a diverse set of cell lines has been undertaken by Holbeck et al. [9] at the National Cancer Institute (NCI) with the screening of the so-called NCI60 panel, a panel of 60 human tumor cell lines (NCI60) representing 9 tissue types, with which about 100,000 compounds and 50,000 natural product extracts have been screened to date. These data are publicly available and have been used here as well.

Further cell lines have been selected to cover a broad set of different indications and according to their response to different standard chemotherapeutics. One aim of this study was to compare the NCI60 panel to our panel of 21 cell lines, including 18 tumor cell lines (786-O, A549, Caco-2, DU 145, HCT 116, HeLa, HT-29, KPL-4, MCF7, MDA-MB-231, MIA PaCa-2, NCI-H460, PC-3, SK-MEL-28, SK-OV-3, SW480, T47D, U-2 OS) and 3 non-tumorigenic cell lines (HaCaT, Hs68, MCF 10A), named hereafter the Bayer Pharma (BPH) panel (see Table 2 and Table 3 for summaries of the respective cell line panels). Cells were obtained from the American Type Culture Collection (ATCC) and the “Deutsche Sammlung von Mikroorganismen und Zellkulturen” (German Collection of Microorganisms and Cell Cultures), (DSMZ) and cultivated in appropriate media according to supplier recommendations. Isolation of genomic Deoxyribonucleic acid (DNA) and total Ribonucleic acid (RNA) was performed using Qiagen systems following provided instructions. Quality of RNA and DNA preparation was analyzed using Agilent Bioanalyser. We deposited the data to the Gene Expression Omnibus (GEO) [10] and are freely available from there (Identifier: GSE41445).

**Table 2:** Cell lines and Tissue of the NCI60 panel (NCI60).

No	CellLine	Tissue
1	786-O	Renal
2	A498	Renal
3	A549	Lung
4	ACHN	Renal
5	BT-549	Breast
6	CAKI-1	Renal
7	CCRF-CEM	Leukemia
8	COLO205	Colon
9	DU-145	Prostate
10	EKVX	Lung
11	HCC-2998	Colon
12	HCT-116	Colon
13	HCT-15	Colon
14	HL-60	Leukemia
15	HOP-62	Lung
16	HOP-92	Lung
17	HS578T	Breast
18	HT29	Colon
19	IGROV1	Ovarian
20	K-562	Leukemia
21	KM12	Colon
22	LOXIMVI	Melanoma
23	M14	Melanoma
24	MALME-3M	Melanoma
25	MCF7	Breast
26	MDA-MB-231	Breast
27	MDA-MB-435	Melanoma
28	MDA-N	Melanoma
29	MOLT-4	Leukemia
30	NCI-ADR-RES	Ovarian
31	NCI-H226	Lung
32	NCI-H23	Lung
33	NCI-H322M	Lung
34	NCI-H460	Lung
35	NCI-H522	Lung
36	OVCAR-3	Ovarian
37	OVCAR-4	Ovarian
38	OVCAR-5	Ovarian
39	OVCAR-8	Ovarian
40	PC-3	Prostate
41	RPMI-8226	Leukemia
42	RXF-393	Renal
43	SF-268	CNS
44	SF-295	CNS
45	SF-539	CNS
46	SK-MEL-2	Melanoma
47	SK-MEL-28	Melanoma
48	SK-MEL-5	Melanoma
49	SK-OV-3	Ovarian
50	SN12C	Renal
51	SNB-19	CNS
52	SNB-75	CNS
53	SR	Leukemia
54	SW-620	Colon
55	T47D	Breast
56	TK-10	Renal
57	U251	CNS
58	UACC-257	Melanoma
59	UACC-62	Melanoma
60	UO-31	Renal

**Table 3:** Cell lines and Tissue of the Bayer Pharma panel (BPH).

No	CellLine	Tissue
1	786-O	Renal
2	A549	Lung
3	Caco-2	Colon
4	DU 145	Prostate
5	HaCaT	Keratinocytes (non-tumorigenic)
6	HCT 116	Colon
7	HeLa	Cervix
8	Hs68	Fibroblasts (non-tumorigenic)
9	HT-29	Colon
10	KPL-4	Breast
11	MCF-10A	Breast (non-tumorigenic)
12	MCF7	Breast
13	MDA-MB-231	Breast
14	MIA-Paca-2	Pancreas
15	NCI-H460	Lung
16	PC-3	Prostate
17	SK-MEL-28	Melanoma
18	SK-OV-3	Ovary
19	SW-480	Colon
20	T47D	Breast
21	U2-OS	Bone

### Microarray Data

The gene expression microarray data for the NCI60 panel have been downloaded from the European Bioinformatics Institute (EBI) ArrayExpress website [11] (Identifier: E-GEOD-32474). Gene expression microarray data for BPH cell line panel has been generated in-house.

The microarray data have been downloaded as CEL-files and have been processed to obtain the gene expression matrix used for modeling, according to the following procedure:

1. CEL-files from real data sets were uniformly processed using the MAS5 algorithm [12] as implemented in the R package *simpleaffy* (version 2.28.0; [13]).
2. All expression values were transformed to log2 values.
3. In case several probe sets shared the same gene symbol, the probe set with the largest mean expression over all samples was used as representative for that symbol.

A subset of 10,846 genes was selected for comparison purposes to already existing studies covering these genes.

Synthetic panel data have been generated according to a previously published procedure [14]. Virtual gene expression values of 1,000 genes have been generated arbitrarily based on 5 state variables for 100 samples. One state variable represents the response to be predicted, called state1. Another response variable, state0, has been constructed by randomly sampling the state1 vector. Consequently, this vector contains no correlation between gene expression and response. State1 represents a positive, state0 a negative control for the computational workflow and thus upper and lower bounds for predictivity.

### Drug Response Data

For the BPH panel, sensitivity towards treatment with eight different chemotherapeutics (Cisplatin, Docetaxel, Paclitaxel, Erlotinib, Gemcitabine, Sorafenib, Regorafenib, Pemetrexed) has been determined by analysis of inhibition of cell proliferation after 72h treatment with different concentrations of respective compounds. The concentration ranges from 1E-05 to 2E-10 mol/L. Cells were seeded into 96-well cell culture plates required to ensure approximately 80% confluence in control at end of experiment. After 72 hours, cells growth was analyzed using Cell-Titer Glow™ according to manufactures instruction. One part of the sample was measured after 24 hours to get “zero-growth” value which corresponds to full inhibition. Data were normalized to Dimethylsulfoxid (DMSO) treated cells as “no-inhibition”. The drug response was quantified by the half maximal inhibitory concentration (IC50) for a particular cell line. IC50 determination was done using a statistical approach based on normalized growth curves of different compound-concentrations.

Correspondingly, drug response data (IC50) of the NCI60 panelfor 48,129 drugs were downloaded from the NCI website [15]. Two subsets have been selected from the available 48,129 drugs from the NCI60 data set.

### Selecting Subsets of Drug Response Data of the NCI60 Panel

One subset contains all drugs that have been tested on the BPH and NCI60 cell line panels (i.e. Cisplatin, Docetaxel, Gemcitabin, Paclitaxel, and Pemetrexed). The second subset is a subset of drugs found by clustering the response values according to the following procedure:

1. Drugs have been removed, if more than five values were missing or less than 29 distinct values existed (13,978 drugs left).
2. Drug response was then standardized for each drug by subtracting the mean value and dividing by the mean absolute deviation.
3. The CLARA clustering algorithm [16], as implemented in the R package *cluster* (version 1.14.2; [17]), has been applied to the preprocessed drug response/cell line matrix. The following parameters have been changed from the default configuration:
  - k = 30 (number of clusters)
  - sample = 50 (number of samples to be drawn from the dataset)
  - samplesize = 500 (number of observations in each sample)
4. The medoids of the fourteen largest clusters have been selected as representatives (NSC numbers: 180973, 18320, 321568, 628115, 679597, 680649, 687350, 687806, 700861, 703472, 710715, 710715, 711816, and 715585).

### Preparation of Gene Expression Data

Different panels of cancer cell lines are commonly available and have been used here to train models for the prediction task. Each panel contains cell lines representing different patients. A matrix of gene expression data X has been constructed for each panel used in this study, as well as a drug response matrix Y that contains individual information of drug efficacy for each cell line.

### Labeling IC50 Measurements

IC50 values were converted to the pIC50 scale (-log10(IC50)). These pIC50 values have been labeled for a binary and a three class classification problem as described in the following.

Labeling for a binary classification problem:

1. Find the class threshold by:
  - Removing of all samples that are equal to the minimum or maximum value of the set.
  - Set the class threshold equal to the median of the remaining samples.
2. Label all samples greater than the class threshold with 2 and the remaining samples with 1.

Labeling for a three class classification problem:

1. Find the two class threshold by:
  - Removing of all samples that are equal to the minimum or maximum value of the set.
  - Set the upper class threshold equal to the 66-quantile of the remaining samples.
  - Set the lower class threshold equal to the 33-quantile of the remaining samples.
2. Label all samples that are greater than the class threshold with 3, the samples smaller than the lower class threshold with 1 and the remaining samples with 2.

The labeling results in three different sets of labels for each drug: The pIC50 values for regression, binary classification and three class classification.

### Utilized Models and their Parameters

#### Lasso (L_1_) regularized logistic regression

We have utilized penalized logistic regression which is a linear model to predict categorical variables. The penalty term used here is called LASSO or L_1_ penalty. The L_1_ penalty term introduces a variable selection of at most n variables, where n is the number of samples available in the training set. A disadvantage in the usage of a penalty term can be observed for group wise correlated variables. In this case, often only one variable per group is selected, while the others are ignored [18].

The implementation used in this study is from the R package *glmnet* (version 1.7.3; [19]). All parameters have been used with default values.

#### Elastic net regularized linear regression

The elastic net penalty term overcomes some of the limitations of the L_1_ penalty. The number of selected variables is not limited. Rather, adding or removing whole groups of variables is encouraged. In addition, the L_1_ regularization path is stabilized [20].

The implementation applied in our study has been taken from the R package *glmnet* (version 1.7.3; [19]). All parameters have been used with default values. The tradeoff parameter has been set to α = 0.5.

#### Support vector machines for regression or classification

A support vector machine (SVM) constructs a hyper plane in a high or infinite dimensional space to separate classes. By maximizing the distance between the hyper plane and the closest samples, intuitively the generalization error is maximized and the risk of overfitting is reduced [21]. SVMs have also been established for binary classification problems in form of a primal optimization problem [22].

We have utilized the implementation from the R package *e1071* (version 1.5–25; [23]). The package provides a wrapper for the LIBSVM library [24]. For classification, the class weights have been set to the reciprocal of the class size. Nested cross validation (i.e. a simple cross-validation where in each validation run, the training set is validated in itself by another split into training and test set) has been used to select tuning parameters from
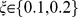
for regression and from
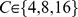
for classification. The SVM types used in this study were *type = eps-regression* and *type = C-classification*.

#### Random forests

The random forest (RF) algorithm is an ensemble method [25] with many decision trees as individual learners. The idea is based on bagging, a method in which each learner is trained on a different bootstrap to increase their variation. The algorithm is described in detail elsewhere [26].

The implementation utilized by us is from the R package *randomForest* (version 4.6-2; [27]) maintaining the default values. The number of trees has been set to 500 for both regression and classification. The number of variables sampled as candidates for each split depends on the problem type. For classification, the number of sampled candidates equals 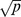 where p is the total number of variables. For regression, the number of sample candidates has been set to *p/3*. The class probabilities have been calculated using the normalized votes of the base learners (trees).

#### Principal component analysis+RF or SVM

The principal component analysis (PCA) has been used as a dimension reduction method, before applying the random forest or SVM algorithm. For each run of the 10-fold cross validation, we have applied the following steps:

1. Train the PCA model on the training data.
2. Use the first five principal components obtained from the PCA model to training the SVM or RF model.
3. Predict the first five principal components for the samples in the test set.
4. Use the PCA prediction as input for the SVM or RF model to predict the classes or regression values.

This way, the instability of the PCA has been included in the cross validation. This would not have been the case, if the PCA would have been calculated on the whole data beforehand. The SVM and RF settings have been chosen as explained above.

#### Two stage random forest

RF has been used to select the most important genes. These genes have then been used to build the final model. To avoid a feature selection bias, the following steps have been executed for each run of the 10-fold cross validation:

1. The random forest has been trained on the training data.
2. The resulting model has been used to rank the genes by the variable importance obtained from the out-of-bag (OOB) data. For classification, the *MeanDecreaseGini* has been used for ranking and the *%IncMSE* for regression.
3. In the next step, the 300 highest ranked genes have been used to retrain the random forest on the training data.
4. Lastly, the so retrained random forest has been used to predict the test set samples.

All settings have been chosen the same way as described for random forests above.

### The Null Model

#### Purpose of the null model

To establish if a model is significantly better than expected by chance, a suitable null model is needed. One way to generate such a null model is estimating the model performance after random permutation of the class labels or target values. This approach ensures that the data of the null model are based on a similar distribution as the real model. After establishing this null model, an empirical p-value can be calculated to reject or accept the following hypothesis:

*H0: The classifier can extract information from the data. This means, there is a correlation between the data and the class labels.*

#### Empirical p-values

For our study, we have utilized empirical p-values proposed by Good [28], supplemented with the recommendations by Ojala et al. [29] as a substitute for the closely related Mann-Whitney-Wilcoxon two-sample rank sum test. This test assumes that the prediction errors are independent of each other, which is however not true in the case of cross validation prediction errors.

### Model Evaluation

#### Background

The metric used to quantify the prediction power of a model plays a key role in finding and tuning the best model. The choice of the metric is important, since metrics put unequal importance to different aspects of the model performance; it is therefore problem and data specific. We have used the following metrics for evaluation:

- *Accuracy:* The ratio of all correct (true positive and true negative) predictions to the total number of cases evaluated (true positive, true negative, false positive and false negative).
  The accuracy metric is not suited, however, for unbalanced classification problems as the contribution of each class depends directly on the number of samples [30]. We therefore calculate additional evaluation metrics:
- *Precision:* The ratio of the number of correct predictions actually made (true positive) to the total number of incorrect and correct predictions (true positive and false positive).
- *Recall:* The ratio of the number of correct predictions actually made (true positive) to the total number of all correct predictions (true positive and false negative).
- *F-Score:* Uses a weighted combination of *Precision* and *Recall* (see above). Further, it is not sensitive to imbalanced classes. The F-Score with β = 1 is called the F1-Score and denotes the harmonic mean of Precision and Recall, used here as defined as follows:

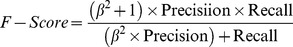



#### Metric based on class probability

Several classifier assign a class probability to each prediction. Precision and recall can be influenced by the choice of a threshold for the class probability. In such a setting, the threshold can be trimmed for varying misclassification costs between the classes. This optimal choice depends on the cost of wrong classification (Precision) versus the cost of missing a sample from a class (Recall). In cases where this tradeoff is not clear, the metric incorporating all possible choices best evaluates a classifier.

Using this receiver-operator-curve (ROC) analysis, the power of the classifier can be defined independently from the decision threshold by calculating the area under the curve (AUC). The AUC is in general better suited to compare classifiers than the accuracy metric [30]. The AUC was originally proposed for binary classifier evaluation but has been extended to multiclass classification tasks as well [31]. We have used both AUC and multiclass AUC for our evaluations.

#### Metric for regression

Regression problems deal with continuous values and are therefore differently evaluated than classification problems.

For this, we have used the Root Mean Square Error (RMSE), measuring the distance between two sets of data points P = [p_1_, …, p_n_] and A = [a_1_, …, a_n_]:
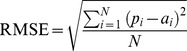



The fact, that the divergence between predicted and observed value is quadratic penalized, makes this metric, however, very sensitive to outliers.

#### Metric use depends on labeling and algorithm

Different metrics have been compared. When assessed as empirical p-values, it can be shown that these metrics are, in general, correlated (data not shown). However, here we report p-values only based upon RMSE (for regression problems) and AUC (two class) or multiclass AUC (tree class problem). SVMs do not provide class probabilities directly, hence there the accuracy metric (two class) resp. F1-score (three class) was used. See Table 1 for an overview of algorithms, labeling and metrics used.

#### Performance estimation for small sample size problems

Independent of the metric used to evaluate the classification or regression model, we have also employed a method to estimate the generalization performance. By this, we mean the expected prediction capability of a model on independent test data. The generalization performance can easily be estimated if a large set of samples is available. In this case, the data are partitioned in a test and a training set. Each set is a representative sample of the overall distribution. The error on the test can therefore be expected to be reliable. One possible split for the two partitions can be such that 70% of the data lies in the training set and 30% in the test set.

In cases of small sample size, however, k-fold cross validation uses the samples more efficiently and provides a more accurate estimate. The data are first partitioned in k folds. Then, each fold is used as test set once, while the model is trained on the remaining data.

Let 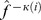 denote the model prediction of the ith sample after having been trained on all data except the kth partition. Here, L denotes the loss function that can be represented by any of the metrics addressed before:
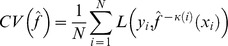



If the model includes a tuning parameter or any kind of feature selection, a second cross validation has to be incorporated in a nested manner (i.e. the training data is partitioned repetitively in inner-training and inner-testing sets).

#### 10x10-fold cross validation

When dealing with microarray data, often very small sample sizes are available (<100). This results in unstable cross validation error estimates that can depend heavily on the partition used for the cross validation. To stabilize the error estimates, the 10-fold cross validation was repeated ten times with different partitions. The results of all ten runs were then averaged. This method, called the 10×10-fold cross validation, is one of the most accurate estimators for direct comparison between models [32].

#### Variations in sample size

The following procedure has been used to examine the influence of sample size on the predictive power of the model. The drugs NSC180973 and NSC700861 have been selected due to their relatively low empirical p-values (see Results). The RF (alone) and PCA+RF algorithms described above have been selected for the evaluation due to their fast execution time. For each drug-algorithm combination, the following steps have been computed:

1. Estimate the RMSE using 10-fold cross validation.
2. Remove five samples and perform the estimation again.
3. Repeat (2) until less than 20 samples remain.

## Results

### Specific Observations

**Figure 2:**
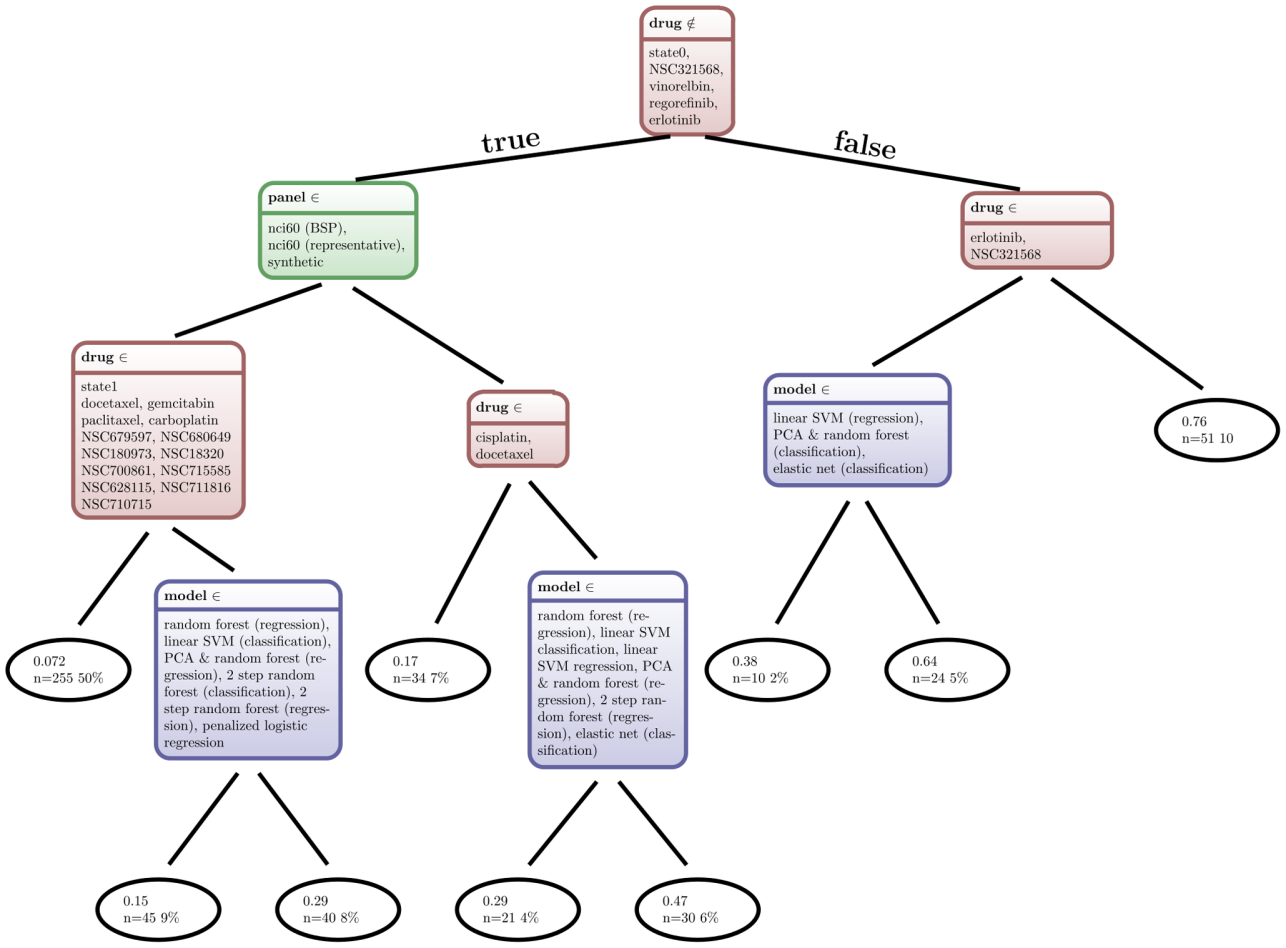
Graphical representation as a decision tree: Factors to achieve good prediction quality. A decision tree has been trained on all empirical p-values obtained from all possible model comparisons. The decision tree has been constructed according to the output from the R package *rpart* (version 3.1–49; [33]), after applying it to the p-value data. Each node represents a decision rule based on one of the four categorical variables: drug, panel, model or labeling. The text in the leaf node gives a summary for each partition with a list of included or excluded entities. The first row of the terminal nodes (elipses) is the mean p-value. The second row displays the number of cases (n), and percentage of total number of cases. The leftmost terminal node represents the lowest achieved p-values, the path through the tree shows the necessary conditions. The *model* is not among them.

The 510 p-values (see Table S1 and S2) have been partitioned into a decision tree by their four categorical variables: drug, panel, model and labeling (see Figure 2). The resulting tree is unbalanced with five nodes and a depth of four on the left side and two nodes and a depth of three on the right side. The left side contains 83% of all samples and the right side the remaining 17%. The factor model occurs three times as split variable. All three splits occur on the lowest split level after a split based on the factor drug. The root node splits based on the factor drug and separates five drugs from the rest. The left side is split on the highest level by the factor panel, which separates the BPH panel samples from the NCI60 and from the synthetic drug (state1– perfect information) samples.

In addition, a random forest model has been trained on the same data as the decision tree (response: p-values, variables: drug/model/panel/labeling). The variable importance based on OOB predictions has been used to rank the results as can be seen in Table 4. Both importance metrics show a clear separation between the two highest ranked factors (compound and panel) and the two lowest ranked factors (model and labeling). This is consistent with the observation from Figure 2, where the root node is split based on the drug factor. The second ranked factor is the panel. The two lowest ranked factors play only a minor role, according to both variable importance measures.

**Table 4:** Variable importance ranking for factors influencing the p-values.

	%IncMSE	IncNodePurity
compound	0.05	15.29
panel	0.03	7.35
model	−8.00E-04	1.77
labeling	−5.00E-05	0.47

The ranking is constructed by random forest on OOB data.

### Correlation between Drug Selection and p-values

**Figure 3:**
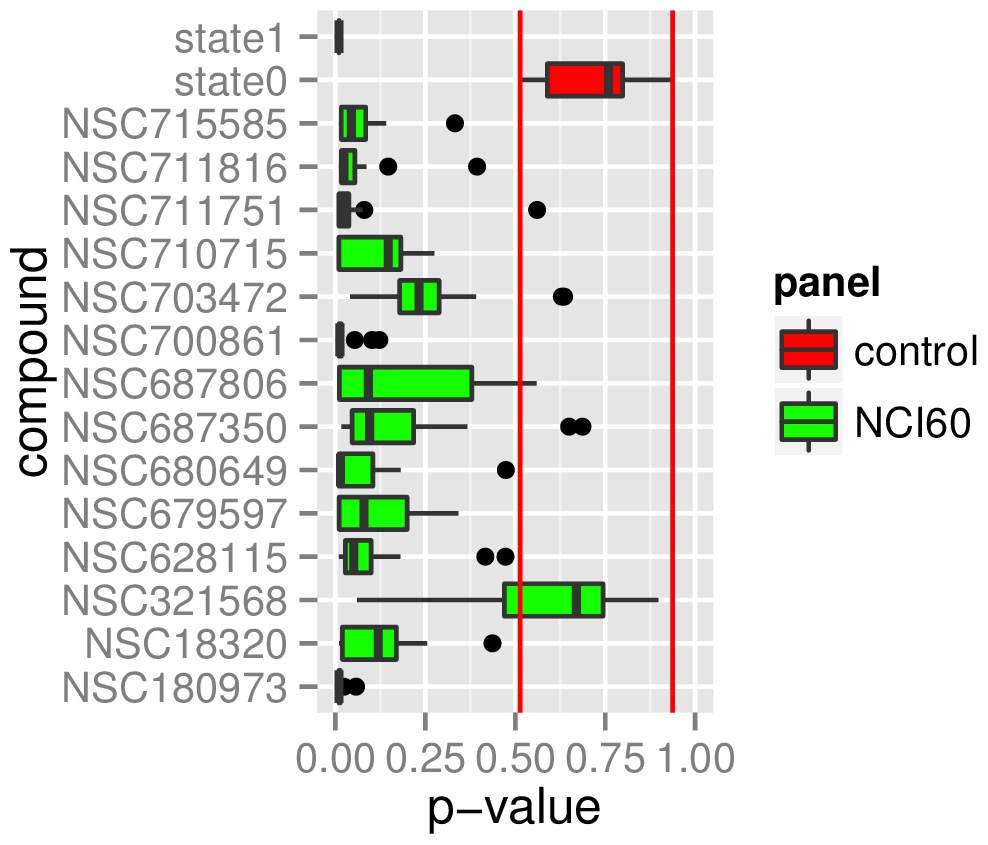
Summary of p-values for NCI60 (representative) and synthetic drug models. Each boxplot summarizes the p-values for all models tested for this drug. The red lines mark the lowest and the highest p-value calculated for the synthetic data with no information.

All empirical p-values for the NCI60 (representative) panel and the synthetic data are summarized in Figure 3 and listed in Table S1. Each boxplot represents all models and labelings that have been tested for this drug. The strong connection between gene expression data and drug response for the simulated drug state1 has been correctly predicted by all models (all models have the lowest possible p-value (p = 0.009)). Two other drugs (NSC700861, NSC180973) have noticeable lower p-values than the others. The simulated drug state0, for which no connection between input and output exists, shows p-values in the range between p = 0.5134 and p = 0.9375– marked by vertical red lines. The median, indicated by the vertical line in the boxplot of the drug NSC321568 is clearly in the range of p-value from state0.

**Figure 4:**
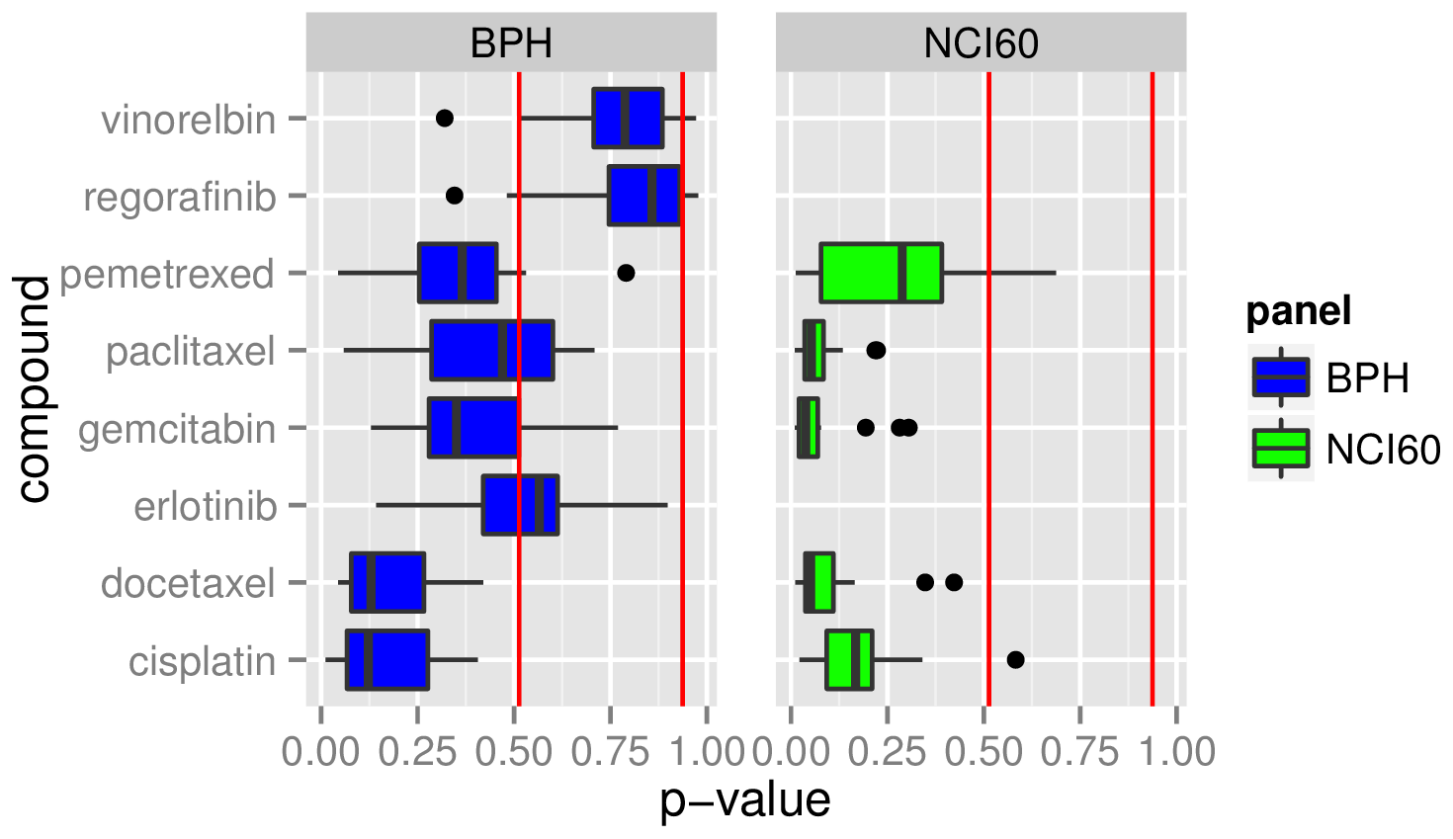
Summary of p-values for BPH and NCI60 models. Each boxplot summarizes the p-values for all models tested for this drug. The red lines mark the lowest and the highest p-value calculated for the synthetic data with no information.

Boxplots for the p-values of the panels BPH and NCI60 are shown side by side in Figure 4. A direct comparison is therefore possible for all drugs that are represented in both panels. The two drugs Vinorelbin and Regorafinib of the BPH panel are clearly not predictable whereas the drugs Docetaxel and Cisplatin have a median below p<0.15. The remaining drugs of the BPH panel show no clear tendency. The drugs in the NCI60 panel have a p-value of p<0.2, with a single exception. The overall picture shows that NCI60 drugs have significantly smaller p-values than the ones from the BPH panel. The p-values for one drug in one panel give no information about the p-values in the other panel.

### Correlation between Panel Selection and p-values

**Figure 5:**
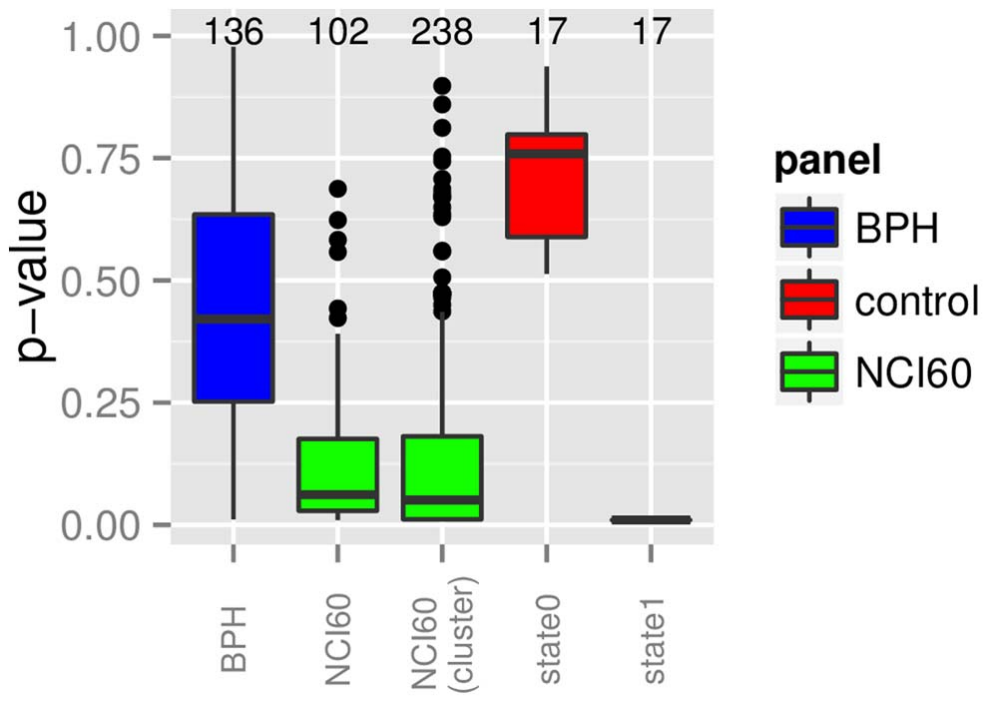
Summary of the p-values for each panel. Each boxplot summarizes all p-values tested on this panel. The horizontal line in the box plot marks the median and the number gives the number of tested models.

Boxplots for all p-values from one panel can be seen in Figure 5. The boxplots for the two synthetic data sets have clearly lower (state1) and higher (state0) p-values than the real datasets. The two subsets of the NCI60 panel are clearly lower than the p-values from the BPH panel. The BPH and the NCI60 panel have eleven cell lines in common. The correlation coefficient has been calculated for five drugs that have been tested on both panels (Table 5). The two drugs Cisplatin and Paclitaxel show a weak correlation (p<0.1) between the panels, while Gemcitabin and Pemetrexed show no correlation. Docetaxel shows no clear sign in either direction.

**Table 5:** Correlation for drug response values between BPH and NCI60 panels.

No.	Cor	p-value	RMSE	n	compound
1	0.54	0.09	0.39	11	Cisplatin
2	0.43	0.18	1.04	11	Docetaxel
3	−0.3	0.37	1.42	11	Gemcitabin
4	0.55	0.08	0.96	11	Paclitaxel
5	0.08	0.82	1.67	11	Pemetrexed

The correlation was calculated for the cell lines that are in both panels.

### Correlation between Model and Labeling Selection and p-values

The two factors model and labeling show no clear connection to the p-values. The choice of the model appears as a splitting variable in Figure 2, but only at the lowest level. The random forest variable importance in Table 4 gives a very low rating to both variables.

### Case Studies

**Figure 6:**
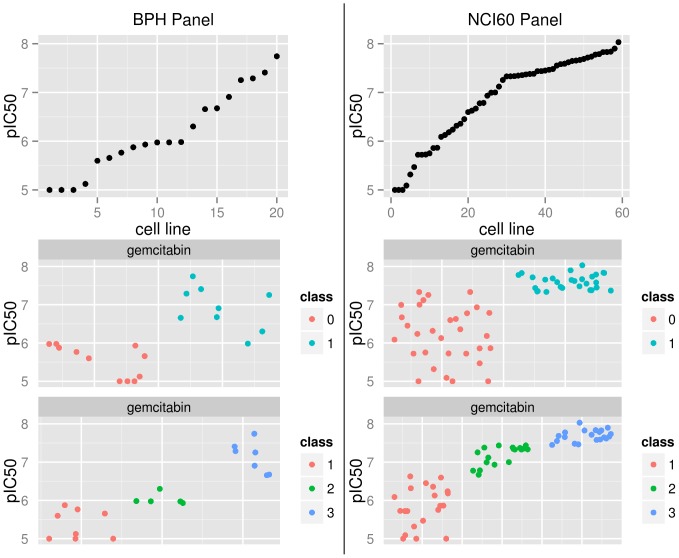
Variation of class threshold between panels for the drug Gemcitabin. The left column is from the BPH panel and the right from the NCI60 panel. The first row shows the unlabeled data, the second and third row color codes labeling for binary and three class labeling. Jitter was added to class labels for display.

Gemcitabin is here used to illustrate the difficulties with class assignments: Figure 6 shows the pIC50 values and the class labels for Gemcitabin for BPH panel (left) and the NCI60 panel (right). The pIC50 values in the first row are sorted by size. The BPH panel contains 19 samples and the NCI60 panel 59 samples. A large proportion of data points are above 7.5 for the NCI60 panel. This influences the class threshold as can be seen in the second and third row. The threshold for the binary labeling is at 6.0 for the BPH panel and at 7.4 for the NCI60 panel. Many samples that would belong to the lower class in the BPH panel are added to the upper class in the NCI60 panel.

**Figure 7:**
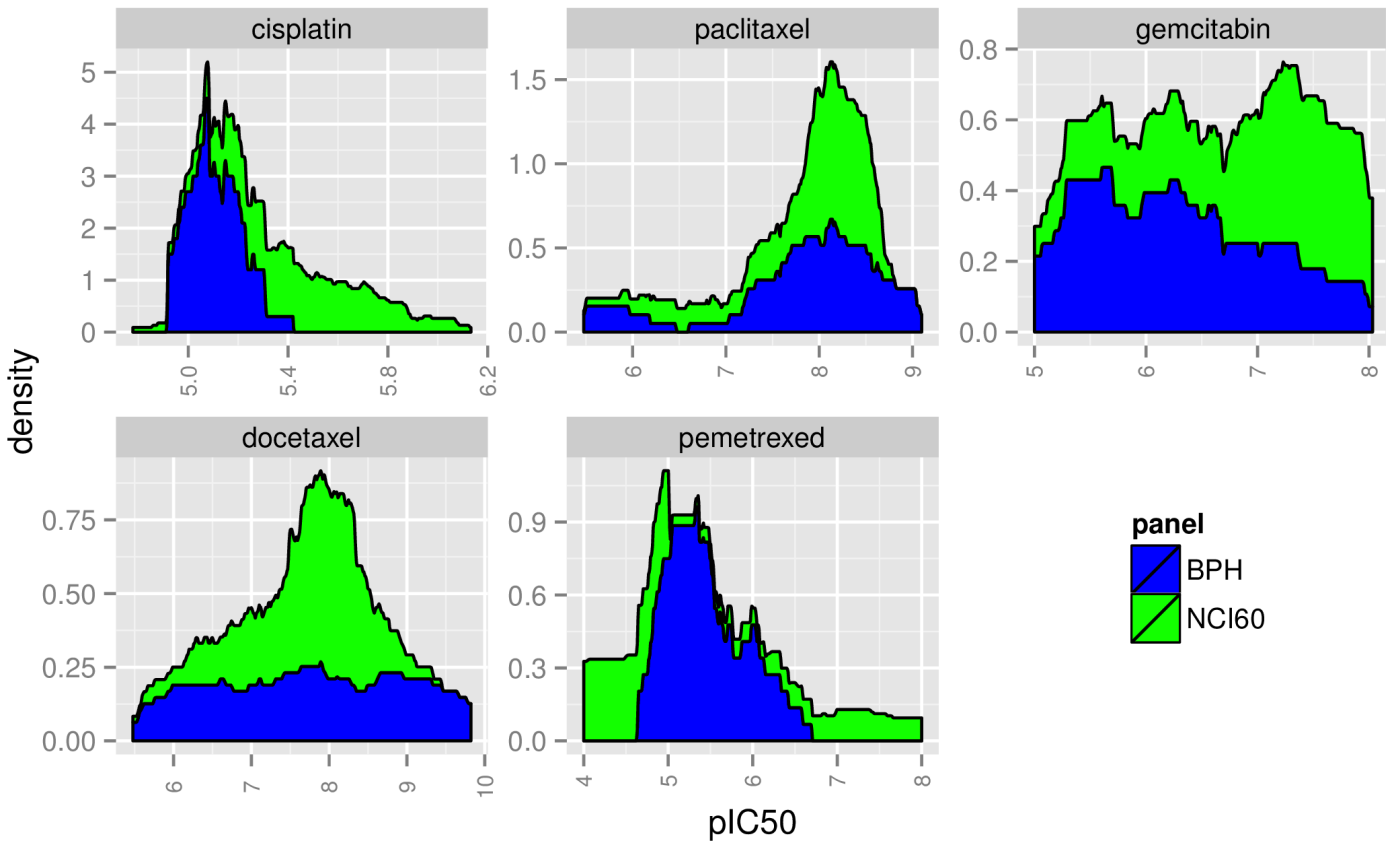
Comparison of drug response value distributions between panels. Overlaid density plots for all drugs that are in NCI60 and BPH panel.

Differences in panels are not only manifested in sample size, but they may, for some drugs, show differences in IC50 value distribution: Density plots for the overlap of drugs between the BPH and the NCI60 panel can be seen in Figure 7. It shows clearly that the drugs Paclitaxel, Gemcitabin and Docetaxel have a similar range on both panels. Pemetrexed, in contrast, has a narrower range on the BPH panel. The range of Cisplatin on the BPH panel covers only the left half of the range on the NCI60 panel. Cisplatin, Pemetrexed and Paclitaxel have a similar shape on both panels. The samples for Docetaxel follow a uniform distribution on the BPH panel but have a peak at 7.9 on the NCI60 panel. Gemcitabin has a similar shape on the left 2/3 of the range.

### Influence of Sample Size on Prediction Error

**Figure 8:**
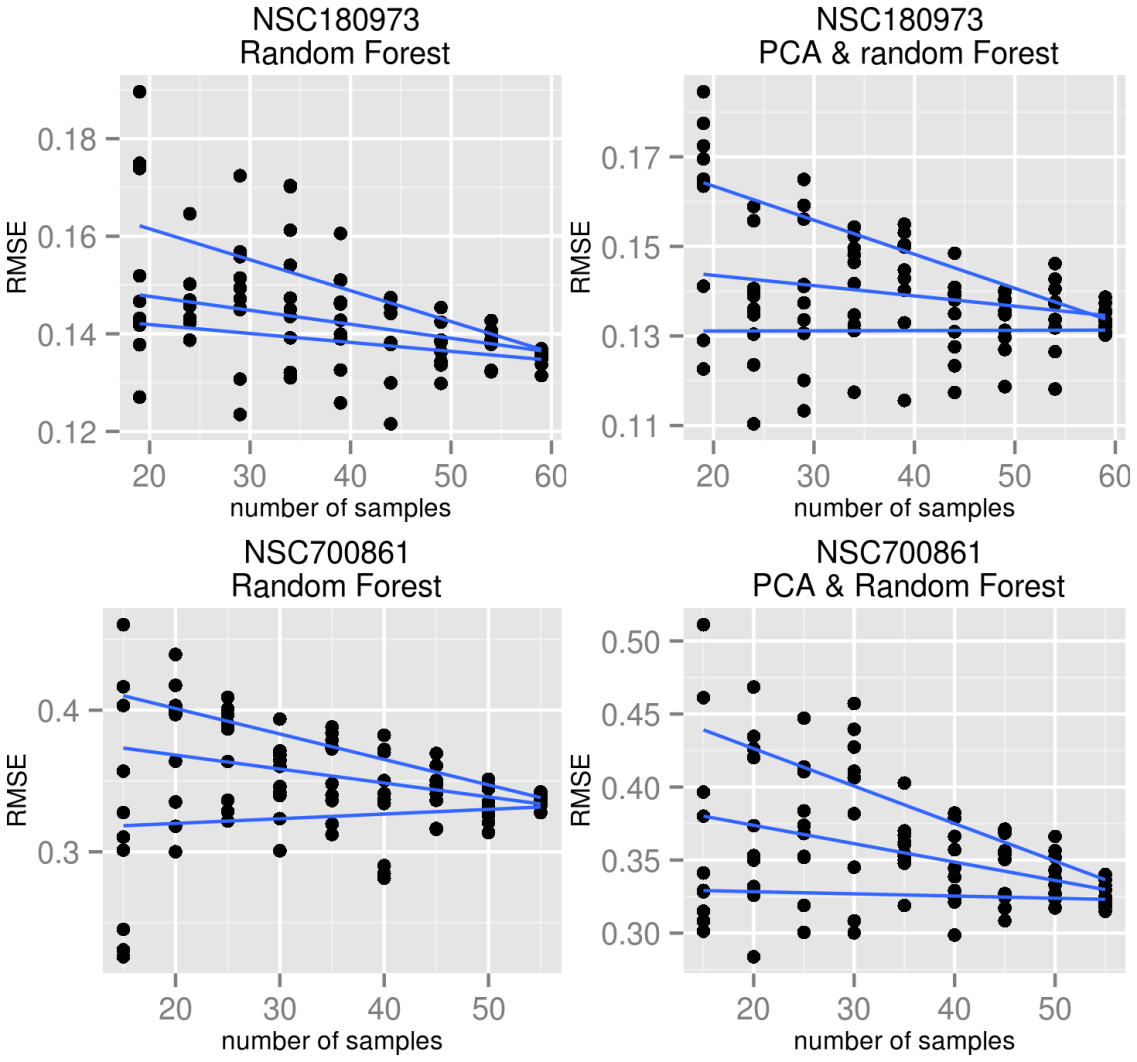
Influence of sample size on prediction error for two selected drugs. The blue lines are the averaged median, upper and lower quartiles. Each dot represents the error estimate of one model evaluation.

Two drugs (NSC180973, NSC700861) have been evaluated for various sample sizes with the random forest algorithm and PCA+RF (see Methods). Those drugs were selected because they are highly predictable (p-value on average 0.01, while the next best predictable compound has an average p-value of 0.03). It can be expected, that even with decreased sample size the data contains sufficient information to train a model. The RMSE and variation of the results decrease with the number of samples used to train the model. This observation is consistent for all four experiments shown in Figure 8. It is of interest to note that the median RMSE increases only slightly with decreasing sample size, while the spread increases strongly.

## Discussion

### Summary

In our present study, we have evaluated drug response predictions for 23 drugs and positive and negative controls. The prediction task has been solved by 17 different machine learning/labeling approaches. The resulting predictions have been analyzed to find factors that influence the prediction quality. It has been found that the choice of the panel and the drug plays a major role for the prediction quality. The two most predictable compounds were used to demonstrate a connection between small sample size and low predictive accuracy. While this is intuitively expected, it is still possible that other algorithms are more robust towards decreasing sample sizes.

The results of this study suggest that the choice of cell line panel and drug have a strong impact on predictiveness, while the selection of machine learning algorithm plays only a minor role in the cases of low predictivity.

We will discuss this in the following with regard to the hypotheses postulated in the Introduction. For evaluation, a decision tree was used to determine the factors leading to low p-values (for the hypothesis that a model predicts drug response) and the irrelevant factors. In addition, a random forest was used to determine the importance of factors towards differentiating between low and high p-values (again, for the hypothesis of model predictivity). To our knowledge, random forests and decision trees have not been used to evaluate results in this way before.

### Hypothesis Concerning the Choice of Drug

#### H1: The successful prediction of efficacy is depending on the particular drug

The first split in the root node of the decision tree (Figure 2) is based on the variable *drug* and, therefore, the information which drug is being predicted by the model. The random forest variable importance supports that the drug selection has a great influence on predictability (Table 4). The boxplots in Figure 3 and Figure 4 show that response to some of the drugs can be predicted reasonably well (i.e. NSC180973 and NSC700861), whereas response to other drugs is essentially unpredictable (i.e. NSC321568). This hypothesis can be accepted based on Figure 3 and Figure 4.

Our finding has several implications. There are drugs that are not predictable by machine learning algorithms which were trained on gene expression data. However, this result could turn out differently when using different cell lines. Therefore, the results should be replicated with different cell line panels to verify this hypothesis further.

If it is confirmed that the *in vivo* response to a specific compound is not predictable from gene expression data, the experimentalist is advised to move to other types of biomarkers.

### Hypotheses Concerning the Machine Learning Methods

#### H2.1: The machine learning algorithms used for the prediction task at hand have a strong influence on the predictability

The random forest variable importance does not indicate that one of the tested algorithms did consistently better than the others. Therefore, this hypothesis is rejected. This is an interesting result, since, on the one hand, algorithms are generally being developed to perform well for a very specific problem type, so that differences in the prediction power of algorithms would be expected. On the other hand, much effort has been put into the selection and comparison of algorithms included into the problem solving model.

One explanation for this result could be the fact, that the problem under consideration is a low predictable task. Therefore, the highly specialized algorithms performed similarly. However, it can be expected that the differences in prediction power between the algorithms become more apparent when the overall predictability of the model is improved. This could be achieved, for example, through a larger sample size or the choice of the panel.

The finding that the choice of machine learning algorithm is of little importance for the overall quality of predictions is interesting and deserves further attention. In particular, it would be of interest if other problems with inherent low predictability would be independent of the choice of algorithms in a similar manner.

For future research in this medical domain, the result could indicate that efforts should be invested in other parameters first, before optimizing the choice of machine learning algorithms. The next hypothesis evaluates the importance of labeling levels.

#### H2.2: One labeling is superior in predictiveness than the others

The random forest variable importance does not indicate that one of the labeling types did consistently better than the others. Therefore, this hypothesis is rejected. The lack of a clear class threshold could have negatively influenced the performance of the classification algorithms. Cisplatin for example has considerable different values on the two different panels (Figure 7, Cisplatin), and therefor different class thresholds. This results in different labeling of one cell line depending on the underlying panel. The regression does not have this restriction since no threshold is needed. Due to that, a regression seems to be the more suite approach for this type of problem.

### Hypotheses Concerning the Choice of Cell Line Panel

#### H3.1: There is a connection between the choice of panel and drug predictability

According to Figure 5, the hypothesis is supported. The predictiveness between the BPH panel and both NCI60 subsets are significantly different. We are currently investigating how much of this is due to the observed variability of the IC50 measurements or the choice of the cell lines in a panel. Figure 7 displays examples ofe compounds that have very different IC50 distributions, which will result in different models.

#### H3.2: The number of samples influences the predictiveness

According to Figure 8, the hypothesis is supported. This could explain the observed differences in predictiveness between the panels. This implicates that for state-of-the-art machine learning algorithms better performance could be expected with larger sample sizes. However, it must be considered that the model selection was only done for the full set of samples and therefor the so found model has been retrained with a decreasing number of samples. This procedure could introduce a selection bias toward the full sample size.

### Caveats

In this study, two independent experimental and an artificial data set was used. While both experimental datasets are different in size, the conclusions drawn from them are similar: Labeling and choice of algorithm are not determining the predictivity. However, this must not be true for all possible data sets.

Data preprocessing and normalization is an important step in data analysis. This step was not varied; hence influence of preprocessing and normalization on the prediction error was not assessed.

In this work, the learning of drug response from gene expression data was studied. Drug response classifier could be trained on different types of data, e.g. genomic data. There, the link between prediction errors and, for example, choice of machine learning algorithm may be different.

### Conclusions and Outlook

This study has shown that the predictability differs between drugs. The machine learning algorithm used for prediction has only a minor influence on this. The choice of the cell line panel, used to obtain the drug response profile and gene expression data, has a strong influence on the predictability.

The recommended approach to identify a predictive model for a new chemical entity is to start with a small panel to assess whether the compound belongs to the cluster of predictable drugs. If that is the case, further efforts shall be directed towards extending this panel until a satisfactory predictability is achieved. The choice of machine learning algorithm is not relevant for the outcome, proficiency should guide the selection. As labeling, regression has some advantages over 2-class or 3-class labeling as for former it is not required to set class boundaries (which depend on previously seen samples) while the quality of prediction is comparable.

It has been shown that quality of drug response prediction depends on the drug under investigation. There is a need to learn more about what causes these differences. Especially the question about biological implications of our findings deserves further attention. The causes for these differences could also be explained by factors that cannot be observed by gene expression data, e.g. structural differences in proteins with a compound specific effect. The results can also be influenced by a lack of diversity in the response or by cell lines that are very sensitive or resistant. In addition, the reliability of the IC50 measurements can be a source of inaccuracy.

We have shown here that the predictability of one and the same drug can greatly differ if different cell line panels are used to obtain the drug response profile and gene expression data. The origin of these differences and whether sample size or other important factors influence these findings is currently under investigation.

## Supporting Information

Table S1 Empirical p-Values for every compound, panel, algorithm, and labeling combination. A comma separated data table: The metric column contains an abbreviation of the used metric, the ‘metric.median’ column has the numerical value in this metric, while ‘p_value’ contains the empirical p-Values estimated from the null-model. Columns expSet, labeling, model, and compound contain the panel, the labeling (one of regression, binary or 3class), the algorithm, and the compound (drug) respectively.
(CSV)

Table S2 Raw prediction quality values for every compound, panel, algorithm, and labeling combination. A comma separated table. Here, the metric used, and the result in each CV run (‘*cv1*’ to ‘*cv10*’) is reported, otherwise columns are labeled as in Table S1.
(CSV)
